# The Role of Mitochondria in Oocyte Maturation

**DOI:** 10.3390/cells10092484

**Published:** 2021-09-19

**Authors:** Anastasia Kirillova, Johan E. J. Smitz, Gennady T. Sukhikh, Ilya Mazunin

**Affiliations:** 1 National Medical Research Center for Obstetrics, Gynecology and Perinatology Named after V.I.Kulakov, of the Ministry of Healthcare of Russian Federation, 117198 Moscow, Russia; secretariat@oparina4.ru; 2 Center of Life Sciences, Skolkovo Institute of Science and Technology (Skoltech), 121205 Skolkovo, Russia; 3 Follicle Biology Laboratory (FOBI), Vrije Universiteit Brussel, 1050 Brussels, Belgium; johan.smitz@uzbrussel.be

**Keywords:** oocyte maturation, mtDNA copy number, mitochondria distribution, IVM, mitochondrial supplementation reagents

## Abstract

With the nucleus as an exception, mitochondria are the only animal cell organelles containing their own genetic information, called mitochondrial DNA (mtDNA). During oocyte maturation, the mtDNA copy number dramatically increases and the distribution of mitochondria changes significantly. As oocyte maturation requires a large amount of ATP for continuous transcription and translation, the availability of the right number of functional mitochondria is crucial. There is a correlation between the quality of oocytes and both the amount of mtDNA and the amount of ATP. Suboptimal conditions of in vitro maturation (IVM) might lead to changes in the mitochondrial morphology as well as alternations in the expression of genes encoding proteins associated with mitochondrial function. Dysfunctional mitochondria have a lower ability to counteract reactive oxygen species (ROS) production which leads to oxidative stress. The mitochondrial function might be improved with the application of antioxidants and significant expectations are laid on the development of new IVM systems supplemented with mitochondria-targeted reagents. Different types of antioxidants have been tested already on animal models and human rescue IVM oocytes, showing promising results. This review focuses on the recent observations on oocytes’ intracellular mitochondrial distribution and on mitochondrial genomes during their maturation, both in vivo and in vitro. Recent mitochondrial supplementation studies, aiming to improve oocyte developmental potential, are summarized.

## 1. Introduction

In vitro maturation (IVM) technology is one of the most promising artificial reproductive technologies, as it allows fertility specialists to obtain oocytes without any or minimal hormonal stimulation on any day of the menstrual cycle [1]. It is mostly suitable for patients with polycystic ovarian morphology, oncological patients, or women with resistant ovary syndrome. This technology has finally surpassed its experimental status [1]; however, the clinical outcomes of the standard IVM programs are still inferior to the control ovarian stimulation programs [2]. It is believed that IVM oocytes have a decreased developmental potential due to cytoplasmic immaturity [3]. As soon as a cumulus–oocyte complex is released from a follicle, meiosis resumes. Yet, at the same time, the oocyte loses gap junctions with the cumulus cells and, therefore, supplementation with nutrients and signaling molecules stops, which leaves the cytoplasm immature. This means that the oocyte’s organelles have a structure and localization typical for the germinal vesicle (GV) stage of development.

One of the main cytoplasmic organelles are mitochondria, which are the source of energy production for the cells and control intracellular Ca^2+^ homeostasis [4,5]. In addition to their importance in energy production and calcium homeostasis, mitochondria also play a central role in many other essential functions of cells, including the regulation of cell death and signaling pathways, iron metabolism, and biosynthesis of some organic compounds [6,7,8]. During folliculogenesis, follicles undergo major growth, expanding approximately 500 times in size. The oocyte within the follicle also undergoes major structural and biochemical transformations and has to complete two meiotic divisions. Naturally, along with this energy-demanding process, the quantity and quality of mitochondria as well as their distribution pattern in an oocyte change [9].

Correct mitochondria functioning is essential for proper oocyte maturation and competence acquisition. Most likely suboptimal in vitro conditions might lead to mitochondrial dysfunction, decreasing IVM oocyte development. In order to determine the factors influencing the mitochondrial pattern during IVM, it is necessary to study the function and dynamics of mitochondria during oogenesis in vivo.

In this review, we will address the complex function of mitochondria in oocyte maturation, discuss the influence of in vitro conditions on the mitochondria, and the strategies for enhancing mitochondrial function during IVM.

## 2. Mitochondrial DNA Biology in Brief

Mitochondria are the only animal cell organelles, except for the nucleus, with their own genetic information, called mitochondrial DNA (mtDNA). The mtDNA is a double-stranded, circular, 16,569 bp DNA molecule in humans, which codes 13 essential subunits of the respiratory chain complexes, 22 tRNAs, and two rRNAs, constituting part of the mitochondrial translation machinery [10]. The mtDNA is stored in cells in nucleoids containing one copy of the genome linked with different nuclear-encoded proteins [11]. The nucleoid seems to be a layered structure in which replication and transcription occur in the central core, whereas translation and complex assembly occur in the peripheral region [12]. The mtDNA has some unique features in comparison to nuclear DNA: (i) multicopy nature of mtDNA with a broad range from several copies in sperm cells [13] to several thousand copies in the mature oocytes [14]; (ii) the mutagenesis rate of the mitochondrial genome is 20 times higher than the mutation rate of the nuclear DNA, with some unique features in its distribution across the genome [15]; and (iii) the multicopy nature of mtDNA allows the existence of mixed populations of mtDNA molecules—a phenomenon called heteroplasmy, when there is more than one mtDNA variant in a cell or an organism [16]. The clinical consequences of mutated mtDNA manifest only once a threshold of the mutant load is exceeded and these functional threshold levels vary, depending on the type of mtDNA mutation [17]. Any mtDNA mutation is supposed to be clinically manifested at the level of 70% heteroplasmy. However, it should be noted that this level is very conditional, and the relationship between the symptoms and the heteroplasmy level is very individual [18,19]. The heteroplasmy level can fluctuate from generation to generation. Hence, women harboring heteroplasmic mtDNA mutations can transmit a wide range of heteroplasmy levels to different offspring within the same sibship. The phenomenon was first shown by Hauswirth and Laipis on a Holstein cow and her offspring when the mtDNA allele variants rapidly shifted and became fixed in a few generations [20]. This is called a “mitochondrial bottleneck”, and it occurs owing to a profound dilution of mtDNA followed potentially by a selective replication of mtDNA genomes and asymmetric segregation of mitochondria. There are two hypotheses describing its time interval: (i) bottleneck as a result of the replication of a separate group of mtDNA during postpubertal folliculogenesis; and (ii) bottleneck during embryogenesis, when there is a significant decrease in the number of mtDNA copies [21,22,23,24].

## 3. Mitochondrial Dynamics in Oogenesis

Structural and morphological studies describe mitochondria from the primordial follicle stage to mature oocytes as naive, roundish-oval-like structures. Unlike mitochondria in differentiated somatic cells, which are mature and form highly structured networks, mitochondria in oocytes have unstructured cristae with a limited capacity for energy production [25]. Measurements of the total number of discrete mitochondria and of mtDNA in mouse oocytes indicate that, on average, each mitochondrion may carry only a single mtDNA copy [26]. To the best of our knowledge, there is no information on the relationship between the mitochondria count and mtDNA copy number in the mature human oocyte, which means that the study reported by Piko and Matsumoto [20] is the only one; everyone takes for granted the extrapolation from a mouse model to human oocytes. If the hypothesis is correct, then the defect mtDNA equals the defective mitochondrion, and an oocyte permits the quality control at the single-genome level by means of selective mitophagy. On the contrary, somatic cell mitochondria are assembled in networks (mitochondrions), and at each moment in time, an individual mitochondrion contains several nucleoids. This “dilution” of pathogenic mtDNA, together with functional mitochondrial complementation, makes the process of recognizing defective mitochondria less efficient in somatic cells [27].

**Figure 1:**
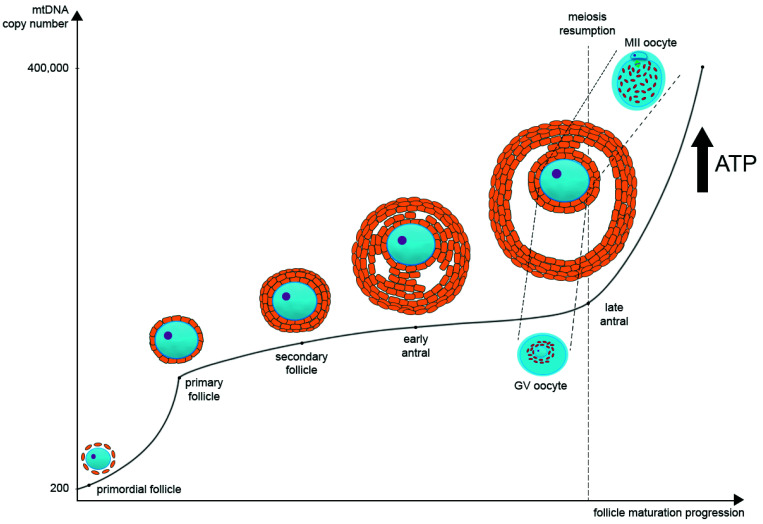
The regulation of mitochondrial DNA (mtDNA) copy number and mitochondria distribution during oocyte maturation. In primordial germ cells, the amount of mtDNA is very low. As oogenesis progresses, mtDNA copies increase significantly and then reach their maximum at the MII stage. There are two main points at which mtDNA copies go up very quickly: the first one is during the transition from the primordial to the primary follicle stage, and the second one is during the transition from GV to MII oocytes at the late antral follicle stage. In parallel as oogenesis progresses, ATP level also increases significantly as oocyte maturation requires a large amount of ATP for the subsequent transcription and translation. During the transition from GV to MII oocytes at the late antral follicle stage, the distribution of mitochondria changes significantly. They migrate from the center of the ooplasm to the pericortical region, distributing evenly through the whole ooplasm.

During oocyte maturation, mtDNA copy number dramatically increases. Most likely, there are two turning points in the mtDNA copy number timeline on the segment from the primordial follicle to the MII oocyte. The first one is during the transition from the primordial to the primary follicle stage, and the second one is during the transition from GV to MII oocytes at the antral follicle stage. Primordial germ cells contain approximately 200 copies of mtDNA, while a mature oocyte possesses about 400,000 copies [28,29] (Figure 1). As noted earlier, such a significant increase in the mtDNA copies during postpubertal folliculogenesis may be one of the bottlenecks of mtDNA inheritance.

In recent years, we have begun to recognize the link between mtDNA replication and mitochondrial fission, which had previously been studied separately [30]. When mtDNA replication terminates, the molecules have to disconnect and separate into different mitochondria. It is known that nucleoids are fixed on the inner mitochondrial membrane (IMM), which helps in the separation of the last ones into different mitochondria during organelle division [31]. The exact protein composition of IMM and the nucleoid interaction site, as well as the detailed mechanism of the interconnection, are still to be recognized. However, it was shown that the loss of the mitochondrial contact site (MICOS) protein Mic60 results in disorganization of both the mitochondrial cristae and nucleotides [32]. It is possible that MICOS may have a role in anchoring mtDNA on the IMM surface in order to prevent their free diffusion around the separating mitochondria. Moreover, both ATAD3 and OPA1 were also colocalized with nucleoid components and have been proposed to be involved in the attachment of mtDNA to the IMM [33,34]. It is an open question whether the association of mtDNA with the IMM is stable or whether mtDNA attaches and separates from the membrane during DNA replication.

It still remains elusive as to which event, nucleoid replication or mitochondria fission, is primary. Mitochondrial morphology is coordinated with the cell cycle and promotes equal segregation of mitochondria during somatic cell division [35]. At the G1 stage of the somatic cell cycle, mitochondria have different morphologies. During the transition between the G1 and S, mitochondria start fusion, likely to enhance the production of ATP required for the S stage and the replication of nuclear DNA in particular. At the G2 and M stages, mitochondria start fission and look like individual organelles distributed throughout the cell [36]. In that condition, mitochondria are distributed between daughter cells. As oogonia cells divide by mitosis, the processes of mtDNA replication and organelle division are most likely similar to somatic cells. After oogonia become primary follicles, the mitotic division stops and the oocytes arrest at the diplotene stage of prophase I. Later on, in the segment between the primordial and primary follicle stage, an increase in both mtDNA copy number and mitochondria is observed [37]. During prophase I, the pairs of homologous chromosomes come together to form a bivalent, which contains four chromatids. Recombination can occur between any two chromatids within this structure which requires a huge amount of ATP [38]. There is another significant increase in the copy number of mtDNA prior to final oocyte maturation [37,39]. Most likely, such accumulation of mitochondria/mtDNA is necessary for further fertilization and further embryo development.

It is interesting that mtDNA replication events concur with the alterations in DNA methylation throughout oocyte maturation [40,41]. The correlation between the decreased DNA methylation levels at exon 2 in human *POLG* (DNA polymerase γ) and an increased mtDNA copy number for the glioblastoma multiforme cell line HSR-GBM1 and hNSCs derived from the NIH-approved human ESC line H9 was demonstrated [42]. We assume these findings could be extrapolated for oocyte maturation, as well as explain the mtDNA copy number changes. During oogenesis, DNA demethylation is mediated by the ten-eleven translocation methylcytosine dioxygenase (TET) family of enzymes. Interestingly, using the Western immunoblotting of samples from primary neuronal cultures, it was possible to find TET in the mitochondrial fraction [43]. It was also shown that demethylation of mtDNA results in an increase in copy number but not the number of mitochondrial transcripts, at least in glioblastoma cells [44]. Demethylation of nuclear and mtDNA likely occurs synchronously, but with different outcomes. DNA demethylation of nuclear-encoded mtDNA replication genes mediates their increased expression, whereas demethylation of the mitochondrial genome provides more templates for mtDNA replication. No changes in the number of mitochondrial transcripts would mean that no additional respiratory chain complexes anchor on the cristae, as mtDNA encodes the core parts of the oxidative phosphorylation complexes. This in turn contradicts the speculation that mitochondria in the oocyte play a key role in supplying the oocyte with ATP and indicates that mitochondria are only a means of mtDNA transmission to the next generation.

As nuclear maturation occurs in the oocyte, the distribution of mitochondria changes significantly. This distribution is very structured rather than chaotic [25,45,46]. According to the data using real-time confocal imaging, a transmission electron microscope, and ultrastructural analysis in in vitro matured oocytes, starting at the GV stage of an antral follicle and until 5 h before the germinal vesicle breakdown (GVB), mitochondria preferentially accumulate close to the perinuclear region and occupy about 80% of the cytoplasm. Then, after GVB and up to the MII stage, mitochondria are distributed equally in the ooplasm and occupy up to 90% of its volume [25,46]. It was shown in a mouse model that during meiosis I mitochondria localize near the spindle and then migrate with pairs of homologous chromosomes towards the pericortical ooplasm. As cytokinesis during meiosis is unequal, most mitochondria are moved back to the spindle poles and are excluded from the polar body 1 (PB1). The same mechanism happens with the extrusion of the second polar body (PB2) [47].

Different subpopulations of mitochondria can be distinguished by considering membrane potential within the oocyte. Highly polarized mitochondria exclusively occupy the pericortical region of the ooplasm, likely of importance to subsequent sperm penetration. Moreover, mitochondria can also differ in size and even nucleoid context within an oocyte [48,49]. Yet, the functional differences in mitochondrial subpopulations still remain to be determined. Furthermore, we believe that results obtained with fluorescent mitochondrial membrane potential probes should be dealt with using great caution. This kind of experimental data requires multiple controls and appropriate interpretation as non-protonic charges may have an effect on the dye behavior [50].

## 4. The Influence of Mitochondrial Function on Oocyte Quality

Female mammals at birth carry millions of primordial follicles containing oocytes arrested at the diplotene stage of prophase I, which constitute the follicular reserve of a female. As oocyte maturation requires a large amount of ATP for continuous transcription and translation, the availability of a sufficient number of functional mitochondria is crucial. However, since the mitochondria of immature oocytes are naive, it is likely that the energy to support oocyte maturation is provided mainly by the surrounding cumulus and granulosa cells [51]. Ovulating oocytes lose their connections with the cumulus cells that have provided them so far with energy and have to activate their own mitochondria. Thus, by the time of final oocyte maturation, a sufficient number of mitochondria have been accumulated. Deviation in mtDNA is associated with ovarian reserve reduction. A distinct range of oocyte mtDNA copy numbers were shown in patients with diminished ovarian reserve (100,000 ± 99,000 copies) compared to women with the normal ovarian reserve (318,000 ± 184,000 copies) [52]. The same pattern was also shown for the polar body mtDNA copy number [53]. These data indicate the importance of the number of mitochondria and mtDNA for oocyte quality.

Meiosis occurs twice during oocyte maturation, once during ovulation (meiosis I), and once during fertilization (meiosis II). Spindle microtubules are assembled from the opposite spindle poles and this process is extremely energy-intensive for oocytes in terms of ATP. There is evidence that decreases in ATP level result in meiotic errors that could alter the number of chromosomes in cells and thus lead to genetic disorders [38,47]. Such errors result in non-extrusion of the PB1, irregular distribution of chromosomes, and aneuploidy. Using HeLa and MDAMB 435 lines as a model system, it was shown that mitochondrial function is fundamental for maintaining the integrity of the genome by preventing chromosomal translocations and rearrangements [54]. Moreover, the correct spindle formation and the occurrence of chaotic mosaicism in human preimplantation embryos are in direct correlation with the value of the mitochondrial membrane potential [55]. ATP levels increase during the polar body extrusion and higher amounts of ATP correlate with a higher fertilization rate of mature oocytes [56].

Mitochondria not only regulate the energy metabolism but also the maintenance of the intracellular Ca^2+^ ion homeostasis [57]. The increasing Ca^2+^ concentration can disrupt oxidative phosphorylation and redox homeostasis or can result in the opening of the mitochondrial permeability transition pore which impairs the mitochondrial function and can cause apoptosis [58]. The low transfer of Ca^2+^ from the endoplasmic reticulum (ER) to the mitochondria contributes to the bioenergy crisis. In turn, disruption of mitochondrial oxidative phosphorylation (mPTP) and ATP production can affect the ATP-dependent calcium fluctuations. The entry of Ca^2+^ into mitochondria is stimulated by the activity of the respiratory chain complexes and causes the movement of protons into the intermembrane space, increasing the potential of the mitochondrial membrane [59].

mtDNA mutations can also result in disease and aging [60]. The study of point mutations and deletion of mtDNA in the oocytes of infertile or aged women has also been carried out. It has been shown that the number of de novo mtDNA mutations in children increases with maternal age [61,62], which might be attributed to oocyte aging. We have carefully estimated the mitochondrial mutation spectrum in offspring and mothers with a reconstruction of the de novo mitochondrial mutations that occurred in oocytes [15], and found that all the analyses of de novo mutations in mother–offspring pairs showed a trend of A > G rate increasing with oocyte’s time of dormancy. Thus, the primary oocyte arrested at the diplotene stage of prophase I can undergo accumulation of mtDNA rearrangements and accumulation of mutations during the long period of dormancy, even for several decades. Such mutations are asymmetric and likely are caused by the oxidation of mtDNA while the oocyte is waiting for fertilization [15]. It has been suggested that primary oocytes carrying deleterious mtDNA mutations are eliminated by follicular atresia, which may explain some cases of diminished ovarian reserve [63].

The purifying selection of pathogenic mtDNA during oocyte maturation is vitally important for offspring, as it can eliminate severe mtDNA mutations [64]. However, the current evidence for germline selection at the meiotic level comes from the incomplete sequencing of mtDNA in the first polar body. Interestingly, new data obtained from PB1 mtDNA deep sequencing revealed a high number of pathogenic mtDNA variants in comparison with its oocyte complement [65]. In order to further explore the transmission of defective mitochondria through generations, Sha et al. transferred the defective mitochondria in mouse germline, including the cumulus–oocyte complexes at the germinal vesicle and MII oocyte stages. The experiments confirmed that during the first and second meiosis, defective mitochondria are moved into PB1 and PB2. It was concluded that the two meiotic processes function as a kind of ultimate tier protection mechanism for future generations [65].

Thus, mitochondrial activity is a balance between the absolute mtDNA copy number, the number of mitochondria in a cell, functional capacity (mutational load), and organelle mobility.

## 5. Mitochondria Patterns during IVM

During IVM, the oocytes are exposed to in vitro conditions which alter the pattern of mitochondria localization and function. Mitochondria have an ability to migrate to areas of high energy consumption, which is crucial for correct oocyte maturation [66]. For instance, the peripheral localization of mitochondria might be necessary near gap junctions which are essential for the two-way communication with the cumulus cells. However, upon maturation, the mitochondria migrate towards the central region, and homogeneous localization of mitochondria is believed to be a sign of cytoplasmic maturity, while the peripheral localization is more common for meiotically incompetent oocytes [67]. In contrast to in vivo matured oocytes, it was demonstrated that IVM oocytes had a tendency of having mitochondria localized more abundantly in the peripheral region than in the inner cytoplasm [68].

Moreover, it was shown that not only the localization but also the functional structure of mitochondria with other organelles might be altered in IVM oocytes. In in vivo matured oocytes, mitochondria form aggregates with tubuli of smooth endoplasmic reticulum (SER) called M-SER, or complexes with small vesicles (0.3–0.5 mm in diameter) called MV [69,70]. However, it was demonstrated that IVM oocytes rarely possess M-SER aggregates but have large MV complexes (up to 2–2.5 mm in diameter) instead [70].

M-SER aggregates are believed to be precursors of MVs [71,72] and the number of M-SER reaches its maximum before ovulation [73]. Most likely, during IVM, the SER tubuli and vesicles are assembled into large MV complexes without M-SER formation. It is possible that suboptimal in vitro culture conditions might lead to the shifting of M-SER to MV complexes, which might lead to lower oocyte competence. As the energy released during M-SER to MV transition might be used for restoring the membranes during cleavage [71], the absence of such a transition during IVM can lead to the decreased developmental potential of the embryos.

Though the number and ultrastructure of mitochondria do not differ between in vitro and in vivo matured oocytes [70], mtDNA copy number has not been compared between the groups. Yet, data on a murine model demonstrated that IVM oocytes had a lower mtDNA content, which might be a sign of inadequate mitochondrial biogenesis or cytoplasmic maturation [74,75]. Nevertheless, the function itself of the mitochondria most likely is not severely impaired due to in vitro conditions. The mitochondrial membrane potential and the general ATP content did not differ for in vitro and in vivo matured oocytes in mice [75]. However, the single-cell transcriptomic analyses comparing in vitro and in vivo matured human oocytes revealed a number of gene pathways associated with mitochondrial function to be different between the groups [76]. For example, 52 genes involved in the calcium pathway which control ER and mitochondria function were altered in the IVM group. Furthermore, there was the abnormal expression of genes involved in the Krebs cycle (ACAT1, HADHA, SDH, and DPYD) in IVM oocytes. ACAT1 and HADHA play a role in mitochondrial b-oxidation, DPYD is an NADP^+^-dependent enzyme, and SDH is an enzyme complex that is bound to the inner mitochondrial membrane of mammalian mitochondria participating in both the Krebs cycle and the electron transport chain [76]. However, as a compensatory mechanism, the activity of nicotinamide nucleotide transhydrogenase was enhanced, allowing the transfer of electrons between NADPH and NADH for the membrane potential generation.

Though it is clear that in vitro conditions might influence mitochondrial patterns in the maturing oocyte, the degree of alterations might depend on the specific culture conditions and the state of cumulus–oocyte complexes themselves. For instance, it was demonstrated on porcine and equine models that the mitochondrial distribution in an oocyte depends on the cumulus morphology [77,78]. COCs with tight cumulus cells had a homogeneous distribution of mitochondria in an oocyte, while COCs with expanded cumulus showed granular or clustered mitochondria patterns [77], which is a trait of cytoplasmic maturity. It is not clear how the IVM medium influences the mitochondria pattern in oocytes. A study in the bovine model demonstrated that the gonadotropin dose does not influence the distribution patterns of mitochondria in IVM oocytes [79]. There is a large diversity of IVM protocols in use and different media and conditions might influence mitochondrial configuration to a different degree. It was demonstrated that high oxygen concentrations (20%) result in a higher embryo yield after IVM in bovine compared to low concentrations (5–7%) [80,81]. However, supraphysiological oxygen levels lead to the increased risk of reactive oxygen species (ROS) formation, which in its turn might compromise mitochondrial function. The impact of different levels of oxygen during in vitro culture in artificial reproductive technologies is, however, unknown [82]. Likely, the composition of the culture media can contribute to the level of superoxide production [82].

IVM systems in humans have been researched and improved over recent years. A promising approach is a biphasic IVM system, using a capacitation culture of 24 h before inducing the oocytes to mature (CAPA-IVM) [83]. This approach produced higher embryological and clinical outcomes compared to “standard IVM”, where COCs are immediately placed in maturation media after retrieval [84,85,86]. The CAPA-IVM system allows for a more physiological cytoplasmic maturation by preserving the gap junctions and allowing oocytes, and fosters cumulus cell communication [83,87,88]. It will be of great interest to study the mitochondrial distribution and function in CAPA-IVM oocytes as well.

## 6. Supplementation of IVM Media with Substances Enhancing the Mitochondrial Function

Though reactive oxygen species are the natural products of oocyte metabolism, the supraphysiological levels of ROS can lead to oxidative stress. Oxidative stress can severely harm the oocyte’s function by oxidating RNA, DNA, and proteins; damaging the cell membrane’s integrity; and shortening the telomeres [89]. The exact mechanism of superoxide production by mitochondria in an oocyte is still unknown [82]. It is believed that impairment of mitochondrial function can lead to excessive superoxide production; however, high ROS levels lead to mitochondrial dysfunction as well.

In vitro gamete manipulations increase the levels of ROS by either direct exogenous ROS production influenced by environmental factors or by indirect intracellular ROS production in response to stress [89]. Antioxidants are molecules that have the ability to counteract the high levels of ROS, thus improving cellular function. Several supplements for the IVM culture have been proposed in order to enhance the mitochondrial function in in vitro matured oocytes in order to increase the subsequent competence of the oocytes and the embryos (Table 1). Interestingly, the majority of the tested IVM supplements’ action is based on the upregulation of the Sirtuin1 (SIRT1) function. SIRT1 is an NAD^+^-dependent deacetylase that increases mitochondrial activity [90,91] and most likely enhances both the biosynthesis and degradation of mitochondria by mitophagy in oocytes [92].

One of the most commonly used antioxidants that regulates SIRT1 function is melatonin (N-acetyl-5 methoxytryptamine). One of the main advantages of this supplement for IVM culture is that it is naturally secreted by cumulus cells [93]. Studies on human oocytes demonstrated that melatonin can improve mitochondrial function by decreasing excessive Ca^2+^ levels and maintaining mitochondrial membrane potential [93]. Supplementation of the IVM culture medium with the follicular fluid-derived melatonin leads to significantly higher implantation rates in PCOS patients [94]. Other studies demonstrated a higher blastocyst formation rate in rescue IVM oocytes [95,96]. Most likely, melatonin supplementation is the most beneficial for the in vitro matured oocytes of older individuals, as it has been demonstrated on a mouse model that the ATP content specifically increases in oocytes from aged animals [97].

Another antioxidant used for supplementation of the IVM system is resveratrol (3,5,40-trihydroxystilbene), which is a plant-based polyphenolic compound [92]. It was proven to enhance the mitochondrial function by increasing the ATP content and mitochondrial membrane potential through the SIRT1 action in animal and human IVM oocytes, leading to higher maturation and blastocyst formation rates [92,98,99]. Moreover, resveratrol was proven to increase the mtDNA copy number and regulates mitochondrial biogenesis and autophagy in bovine IVM oocytes [100]. Supplementation of the medium with 1.0 µm resveratrol resulted in improved spindle morphology and more correct chromosomal localization in human rescue IVM oocytes [92].

*SIRT1* expression can be upregulated via another promising plant-based chemical, the flavonoid quercetin (3, 3′, 4′, 5, 7-pentahydroxyflavone) [101]. This agent has proven to improve IVM outcomes in porcine, goat, mice, and human oocytes by improving mitochondrial function [101,102,103]. The action of quercetin seems to be multidimensional as it prevents the formation of abnormal mitochondrial structure, reduces the levels of oxidative stress, the extent of apoptosis, increases mitochondrial ATP levels, and promotes mitophagy [101]. Similar to resveratrol, the quercetin did not change the mitochondria number; however, it promoted more mitochondria with fewer structural abnormalities. For instance, there were fewer mitochondria vacuoles, swollen vesicles, narrowed inter-membrane spaces, loss of cristae, and myelin figures in the IVM oocytes treated with quercetin [101]. The improved mitochondrial function in human rescue IVM oocytes led to increased fertilization (76.2% vs. 62.2%) and blastocyst formation (33.3% vs. 17.8%) rates, indicating the improved in vitro matured oocytes’ developmental competence in the presence of quercetin [101].

A few other antioxidants in IVM cultures have recently been tested. For instance, supplementation of IVM medium for bovine oocytes with anethole (1-methoxy-4-(1E)-prop-1-en-1-ylbenzene) was proven to result in higher cleavage, embryonic development, and total cell number per blastocyst rates, yet without improving the oocyte maturation rate [104]. The exact mechanism of anethole action is as yet unknown, but it was proposed that it might act as a synergistic antioxidant, increasing the activity of primary antioxidants such as superoxide dismutase, catalase, and glutathione peroxidase [104].

Immature porcine oocytes benefited from the supplementation of IVM culture with the mogroside V, which led to reduced ROS levels in oocytes, increased mtDNA copy number, mitochondrial membrane potential, and ATP generation, and the expression of oxidative stress-related and mitochondria-related genes, thus promoting higher maturation and blastocyst formation rates [105]

Supplementation of the IVM system in animal models with the electron transporter in the mitochondrial inner membrane coenzyme Q10 (CoQ10) resulted in increased mitochondrial function and better embryological outcomes [106,107]. It demonstrated, for the ovine oocytes, the more diffused physiological pattern of mitochondrial distribution, higher mitochondrial membrane potential, and reduced ROS levels in the CoQ10 group [107], as well as higher ATP levels, mitochondrial charge polarization, and greater mitochondrial mass for the bovine oocytes [106]. However, the supplementation of the IVM medium with CoQ10 in a pig model did not result in the improvement of any embryological outcomes [108], likely because porcine oocytes might be less liable to stress during in vitro culture. Nevertheless, inspiring results were obtained in human IVM oocytes. Supplementation of the IVM medium with 50 µmol/L of CoQ10 resulted in 20% increased maturation rates and decreased aneuploidy rates in the first polar body by 28% in older patients [109].

Another promising mitochondrial-targeted agent for IVM culture is the antioxidant mitoquinone mesylate (MitoQ). It has been shown to improve the maturation and embryo development rates for murine and human oocytes [110,111]. Since MitoQ has a lipophilic quinone moiety linked to a triphenylphosphonium (TPP) moiety in its structure [112], it can be effectively taken up by mitochondria without any carrier [113]. Inside mitochondria, MitoQ adsorbs to the inner membrane and acts as an antioxidant against lipid peroxidation [98]. It was shown to increase the mitochondrial membrane potential and decrease the intracellular ROS levels in oocytes during the IVM culture [110,111]. MitoQ seemed to have the most positive effect for aged oocytes, since it has an ability to prevent chromosomal misalignment [111], which is common for females of advanced reproductive age.

Although there is a large amount of accumulated data on the beneficial effect of different mitochondrial-targeted supplements for IVM culture, none have yet been introduced into clinical practice. First of all, it is very important to select the right concentration of the supplement, as excessive concentrations might lead to a toxic, detrimental effect on the oocytes. Secondly, the correct model should be chosen for antioxidant testing. Not all data can be extrapolated from animal models. The only available data on human oocytes currently are on “rescue IVM”, i.e., the surplus immature oocytes from the controlled ovarian stimulation cycles which failed to mature even after the trigger administration. Such oocytes are not representative of oocytes from small antral follicles, as they have a decreased developmental potential and lack the tightly interconnected cumulus cell layers essential for more physiological in vitro maturation. Ultimately, it is of great importance to study the influence of antioxidant supplementation on the health of the offspring in animal models. Nevertheless, the approach of enhancing the mitochondrial function in in vitro matured oocytes seems promising so far. Specifically, women of advanced reproductive age who tend to have dysfunctional mitochondria [114] might benefit from such treatment.

**Table 1:** Summary of the antioxidants applied for IVM culture improvement. IVM: in vitro maturation. ↑: increases. ↓: decreases. ROS: reactive oxygen species. CoQ10: coenzyme Q10. AMPK: AMP-activated protein kinase. mtDNA: mitochondrial DNA. MitoQ: Mitoquinone mesylate. SIRT1: Sirtuin1.

Antioxidant	Model	Effect	Possible Mechanism	Reference
melatonin	mouse oocytes	↑ maturation rate	↓ excessive Ca^2+^ levels upregulate the SIRT1 function ↑ATP generation ↓ROS levels ↑mtDNA copy number	Nasheed Hamad Almohammed et al. 2020 [97]
	human oocytes	↑ implantation rates		Kim et al. 2013 [94]
	rescue IVM oocytes	↑high-quality blastocyst formation rate ↓aneuploidy rate		Zou et al. 2020 [96]
resveratrol	mouse oocytes	↑ maturation, fertilization, and blastocyst formation rates ↓ chromosomal misalignment	↑ATP generation content ↑mitochondrial membrane potential ↑mitochondrial number	Liu et al. 2018 [92]
	bovine oocytes	↑ fertilization and blastocyst formation rates ↑ blastocyst cell number		Takeo et al. 2020 [98] Sugiyama et al. 2015 [100]
	porcine oocytes	↑ blastocyst formation rate ↑ total blastocyst cell number		Saro et al. 2014 [99]
	human rescue IVM oocytes	↑ maturation rate ↓ chromosomal misalignment		Liu et al. 2018 [92]
quercetin	parthenogenetically activated porcine oocytes	↑ blastocyst formation rate	prevents formation of abnormal mitochondrial structure ROS levels ↓ the extent of apoptosis. ↑ATP generation promotes mitophagy	Kang et al. 2013 [102]
	goat oocytes	↑ maturation rate		Silva et al. 2020 [103]
	mouse oocytes	↑ maturation and early embryonic development formation rates		Cao et al. 2020 [101]
	human rescue IVM oocytes	↑ maturation and blastocyst formation rates		Cao et al. 2020 [101]
anethole	bovine oocytes	↑ cleavage and total cell number per blastocyst rates	likely ↑ the activity of primary antioxidants (superoxide dismutase, catalase, and glutathione peroxidase)	Sá et al. 2020 [104]
CoQ10	ovine oocytes	↑ blastocyst formation and hatching rates ↓ chromosomal misalignment	physiological diffused pattern of mitochondrial distribution ↑mitochondrial membrane potential ↓ intracellular ROS levels ↓glutathione levels ↑ATP generation ↑mitochondrial number ↑AMPK activity	Heydarnejad et al. 2019 [107]
	bovine oocytes	↓ oocyte death		Abdulhasan et al. 2017 [106].
	porcine oocytes	no effect		Maside et al. 2019 [108]
	human oocytes	↑ maturation rate ↓aneuploidy rate		Ma et al. 2020 [109]
mogroside V	porcine oocytes	↑ maturation and blastocyst formation rates	↑mtDNA copy number ↑mitochondrial membrane potential ↑ATP generation ↑expression of oxidative-stress-related and mitochondria-related genes ↓ intracellular ROS levels	Nie et al. 2019 [105]
MitoQ	murine oocytes	↑ maturation, fertilization, and blastocyst formation rates	antioxidant against lipid peroxidation ↑mitochondrial membrane potential ↓ intracellular ROS levels	Hosseinzadeh Shirzeyli et al. 2020 [110]; Al-Zubaidi et al. 2021 [111]
	human rescue IVM oocytes	↑ maturation rate ↓ chromosomal misalignment		Al-Zubaidi et al. 2021 [111]

## 7. Conclusions

Mitochondria are found in high numbers in oocytes as they provide energy for its maturation. The mtDNA copy number increases during all stages of oocytes’ growth and maturation, from the primordial follicle stage until the ovulatory antral follicle stage, but there are likely two main points at which copies increase rapidly. In addition, in oocytes, the organelle seems to be naïve, according to its structure and morphological features. The absence of complexly ordered cristae might suggest that mitochondria are not actually the energy factories for the oocytes. There is speculation that mitochondria may ensure only the transmission of the mtDNA between generations, while the recharging of oocytes is carried out by the surrounding granulosa and cumulus cells. Another assumption states that there are several subpopulations of mitochondria, each with its own function: some of them may generate ATP by oxidative phosphorylation, while the others transmit the mtDNA to future generations. Yet, the functional differences in mitochondrial subpopulations remain to be determined. There is strong evidence that the quantity and the quality of mitochondria are vital for the successful maturation of the oocyte, its fertilization, and subsequent embryo formation. The correlation between the quality of oocytes and both the amount of mtDNA and the amount of ATP was also shown. Experiments describing the influence of IVM conditions are still contradictory, but both a change in the expression of gene-encoding proteins associated with mitochondrial function and a change in the mitochondrial morphology have been documented. Significant expectations are laid on the development of new IVM systems supplemented with mitochondria-targeted reagents. Different types of antioxidants have been tested in animal models and in human rescue IVM oocytes, showing promising results. Though the majority of studies have focused on the dynamics and function of mitochondria in human oogenesis, some initial research addressed the implementation of different molecules to increase mitochondrial function. The first results from this work are encouraging; continuation of this work along the same line of thinking is ongoing.
